# Posttranslational regulation of Akt in human cancer

**DOI:** 10.1186/2045-3701-4-59

**Published:** 2014-10-01

**Authors:** Chia-Hsin Chan, Ukhyun Jo, Abraham Kohrman, Abdol Hossein Rezaeian, Ping-Chieh Chou, Christopher Logothetis, Hui-Kuan Lin

**Affiliations:** Department of Pharmacological Sciences, Stony Brook University, Stony Brook, NY 11790 USA; Department of Molecular and Cellular Oncology, University of Texas MD Anderson Cancer Center, Houston, TX 77030 USA; Graduate School of Biomedical Sciences, University of Texas Health Science Center at Houston, Houston, TX 77030 USA; Department of Genitourinary Medical Oncology, University of Texas MD Anderson Cancer Center, Houston, USA; Graduate Institute of Basic Medical Science, China Medical University, Taichung, 404 Taiwan; Department of Biotechnology, Asia University, Taichung, 404 Taiwan

**Keywords:** Akt, Posttranslational modification, K63-linked ubiquitination, Cancer therapy

## Abstract

Akt regulates critical cellular processes including cell survival and proliferation, glucose metabolism, cell migration, cancer progression and metastasis through phosphorylation of a variety of downstream targets. The Akt pathway is one of the most prevalently hyperactivated signaling pathways in human cancer, thus, research deciphering molecular mechanisms which underlie the aberrant Akt activation has received enormous attention. The PI3K-dependent Akt serine/threonine phosphorylation by PDK1 and mTORC2 has long been thought to be the primary mechanism accounting for Akt activation. However, this regulation alone does not sufficiently explain how Akt hyperactivation can occur in tumors with normal levels of PI3K/PTEN activity. Mounting evidence demonstrates that aberrant Akt activation can be attributed to other posttranslational modifications, which include tyrosine phosphorylation, O-GlcNAcylation, as well as lysine modifications: ubiquitination, SUMOylation and acetylation. Among them, K63-linked ubiquitination has been shown to be a critical step for Akt signal activation by facilitating its membrane recruitment. Deficiency of E3 ligases responsible for growth factor-induced Akt activation leads to tumor suppression. Therefore, a comprehensive understanding of posttranslational modifications in Akt regulation will offer novel strategies for cancer therapy.

## Introduction

*Akt*, also known as protein kinase B (PKB), was originally identified as the cellular homologue of the viral oncogene, *v-akt* [1, 2]: a serine/threonine kinase similar to protein kinases A and C [3, 4]. Akt serves as a central hub that transduces various extracellular cues to regulate a wide range of biological processes through phosphorylation of distinct protein substrates. For instance, Akt phosphorylates and inhibits FoxOs, GSK3β, TCS2 and Bad [5]. FoxOs, when phosphorylated by Akt are sequestered in the cytoplasm where they fail to induce transcription of genes associated with apoptosis and cell cycle arrest [6]. Akt phosphorylation of GSK3β, a protein kinase known to downregulate protein substrates for cell survival and proliferation, causes cells to reacquire survival features [7]. TSC2 inhibits mammalian target of rapamycin complex 1 (mTORC1), a protein complex essential for protein translation and cell growth. The phosphorylation of TSC2 by Akt allows the reactivation of mTORC1 [8, 9]. Akt phosphorylation of Bad, a pro-apoptotic protein that controls mitochondrial outer membrane permeability, decreases cytochrome c release to protect cells from apoptosis [10]. Akt-regulated phosphorylation can also provoke the activation of substrates like IKKα and Skp2 [11–13]. Akt phosphorylates and activates IKKα, leading to the induction of immune response through upregulation of NF-κB. Skp2 is an F-box protein constituting the Skp2 SCF E3 ubiquitin ligase complex and is essential for cell cycle progression, migration and metastasis [13–16]. Akt-mediated phosphorylation of Skp2 stabilizes and activates Skp2 and facilitates its cytosolic translocation, thereby promoting cell migration and cancer metastasis [12, 13, 17].

Change in Akt expression level or activity is a causative factor for the onset or progression of a variety of human cancers [18–20]. Therefore, there is great enthusiasm in the scientific community for a better understanding of the regulatory mechanisms that underlie abnormal Akt activation and expression. In fact, Akt exists as three isoforms in mammals [21, 22]. Akt1 is ubiquitously expressed in various tissues at high levels. Akt2 is highly expressed in the muscle, liver and adipose tissues while modestly expressed in other tissues. Akt3 is predominantly expressed in brain and testis. These three isoforms sharing high sequence and structural homology consists of an amino-terminal pleckstrin homology (pH) domain and a carboxyl-terminal regulatory domain. Since these Akt isoforms are similarly activated by PI3K and PDK1 and share some common downstream effectors, it is postulated that the Akt isoforms may be functionally redundant. However, accumulating evidence suggests differential substrate specificity and biological functions among Akt isoforms (reviewed by B. Dummle and B.A. Hemmings) [23]. These observations therefore redirect scientists’ attention to the roles of each Akt isoform in physiological and pathological conditions.

A plethora of downstream players for each Akt isoform have been discovered and characterized [5, 23] and the involvement of many substrates in specific biological functions and diseases has been comprehensively illustrated (reviewed by B.D. Manning and L.C. Cantley) [5, 23]. In this review, we will summarize recent advances in the posttranslational regulation of Akt, discuss the possible roles of Akt regulators affecting Akt posttranslational modifications in cancer development and potentially provide new therapeutic strategies for cancer targeting.

### Phosphorylation and activation of Akt

Extensive studies have established that growth factors, hormones and cytokines stimulate Akt activation through serial phosphorylation events [24, 25]. The binding of ligand to its cognate receptor tyrosine kinase (RTK) causes PI3K activation that converts phospholipid PIP_2_ to phospholipid PIP_3._ PIP_3_ then interacts with and recruits both Akt and phosphoinositide-dependent kinase 1 (PDK1) to the plasma membrane where PDK1 can phosphorylate Akt at threonine (Thr^308^) in the activation loop, consequently keeping Akt in its active conformation [26]. While this phosphorylation is sufficient to activate some Akt downstream substrates, additional phosphorylation in the C-terminal domain at serine 473 (Ser^473^) by mTORC2 accounts for the full activation of Akt [27]. Other than mTORC2, kinases including DNA-dependent protein kinase (DNA-PK), integrin-linked kinase (ILK), and mitogen-activated protein kinase-activated protein kinase-2 (MAPKAPK2) have been implicated in the modulation of Akt phosphorylation at Ser473 in various contexts [28–31]. Most recently, Wei’s group uncovered a novel phosphorylation event that critically regulates Akt activation under diverse physiological conditions. They found that Akt S477/T479 phosphorylation can be triggered by cyclin-dependent kinase 2 (cdk2)/cyclinA, mTORC2, and DNA-PK under cell cycle progression, growth factor stimulation and DNA damage response [32, 33]. While Akt S477/T479 phosphorylation is essential for Akt activation and Akt-regulated biological functions, it remains obscure how this phosphorylation orchestrates Akt activation [32]. It will be interesting to determine whether Akt S477/T479 phosphorylation regulates Akt activation by affecting its membrane recruitment under such physiological cues.

Conversely, phosphatase and tensin homolog (PTEN) is a phosphatase that dephosphorylates PIP_3_ to prevent Akt membrane recruitment and phosphorylation [34]. Multiple lines of genetic evidence demonstrate that PTEN loss results in aberrant hyperactivation of Akt that renders PTEN-deficient mice to predispose to neoplasia and tumors [35]. In addition to PTEN, two phosphatases, protein phosphatase 2A (PP2A) and PH domain and leucine rich repeat protein phosphatase (PHLPP), were found to dephosphorylate Akt at Thr308 and Ser473, respectively, thereby antagonizing Akt activation [36–38].

Besides PI3K and the upstream kinases and phosphatases of Akt, several proteins indirectly involved in cycles of serine/threonine phosphorylation and dephosphorylation of Akt have been identified. For instance, promyelocytic leukemia protein (PML) represses Akt activation as it is essential for PP2A-mediated Akt dephosphorylation at Thr308 [39]. Consequently, genetic ablation of PML causes prostate tumors in mice accompanied by elevation of Akt phosphorylation and activation [39]. In the context of dopamine stimulation, Caron’s group showed that β-arrestin-2 facilitates the interaction of PP2A with Akt, leading to dephosphorylation and inhibition of Akt [40, 41]. Further genetic evidence showed β-arrestin-2 knockout mice exhibit defects in dopamine-mediated neurotransmission and Akt inactivation in the central nervous system [24, 25]. Similarly, FK506 binding protein 51 (FKBP51) works to bridge PHLPP and Akt leading to Ser473 dephosphorylation and subsequent inactivation of Akt [42].

Multiple studies present evidence that Akt undergoes tyrosine phosphorylation in response to diverse growth factors, such as EGF, Heregulin and insulin. Upon EGF stimulation, Akt is phosphorylated at Tyr315 and Tyr326 by Src or protein tyrosine kinase 6 (PTK6), a Src-related tyrosine kinase [43, 44]. Both Qiu’s and Tyner’s groups reported that phosphorylation of Akt at Tyr315 and Tyr326 is required for its kinase activation, as substitution of these residues with phenylalanine abolishes its kinase activity. While Qiu’s study suggests that PI3K activation is essential for Akt tyrosine phosphorylation, Tyner’s work does not support this notion. This discrepancy may be due to the different concentrations of EGF and the different cell types used in their studies. Nevertheless, Luan and colleagues revealed that enhanced Akt tyrosine phosphorylation by β-arrestin-2 can activate Akt signaling. In contrast to its negative role in Akt activation upon dopamine stimulation, β-arrestin-2 facilitates Akt activation by recruiting Akt to active Src and the insulin receptor complex for its tyrosine phosphorylation upon exposure to insulin [45]. In line with this observation, loss of β-arrestin-2 results in defective Akt activation and attenuates insulin signaling, thereby leading to insulin resistance and type II diabetes in mouse models [45]. Altogether, β-arrestin-2 functions as a contextual Akt regulator that can instigate opposing effects on Akt. Thus, caution should be taken when considering β-arrestin-2 as a target for therapeutic intervention.

Akt can also be phosphorylated at Tyr176 by Ack1 upon activation of RTK by growth factors [46]. Mahajan and colleagues have shown that Akt Tyr176-phosphorylation is sufficient to drive its membrane localization and subsequent PDK1- and mTORC2-mediated Akt phosphorylation and activation. Interestingly, the Ack1-mediated Akt tyrosine phosphorylation remains unchanged in the presence of PI3 kinase inhibitor, LY294002. This finding not only lends a support but also offers a molecular mechanism for the previous notion that Akt activation can occur in a PI3K-independent manner [47–49]. Experiments using transgenic mice expressing constitutively active Ack1 in prostate tissues show an elevation of Tyr176-phosphorylation and activation of Akt *in vivo*. Further, these mice develop prostatic intraepithelial neoplasia (PIN) similar to those driven by Akt hyperactivation. However, it is unclear whether Ack1 is required for prostate cancer development upon conditional PTEN inactivation in prostates.

### Lysine modifications (ubiquitination, SUMOylation and acetylation) of Akt

Phosphorylation and dephosphorylation are regarded as the primary mechanism driving Akt signal activation. Recent advances reveal that other posttranslational modifications including ubiquitination and SUMOylation are also essential and equally important as phosphorylation for Akt signaling activation.

Ubiquitination, just like phosphorylation, is a dynamic modification engaged in diverse cellular processes including cell growth and proliferation, apoptosis, DNA damage response, inflammation, cancer and neurodegenerative diseases [50–55]. It is an enzyme-catalyzed cascade that not only marks protein substrates for 26S proteasome-dependent degradation by covalently conjugating them with multiple ubiquitin monomers via lysine (K)48-linkage, but also alters protein localization, trafficking and/or activation via K63-linked polyubiquitin chains [56, 57]. The vital role of ubiquitination in regulating Akt degradation and inactivation has been recently defined. For instance, CHIP, BRCA1, TTC3, and MULAN ubiquitin ligases have been shown to promote K48-linked ubiquitination and degradation of Akt to terminate its activation [58–61].

In 2009, our group presented the first evidence that Akt can undergo K63-linked ubiquitination at K8 and K14 residues within its PH domain in response to IGF-1 and cytokine IL-1 stimulation. In contrast to K48-linked ubiquitination and degradation, this K63-linked ubiquitination does not trigger Akt degradation. Instead, it is required for Akt membrane recruitment, serine/threonine phosphorylation and activation [62]. TRAF6 was the first example of an E3 ligase that orchestrates Akt signaling activation by promoting its K63-linked ubiquitination [62]. In line with this study, a later report showed that Nedd4 is another E3 ligase which regulates K63-linked ubiquitination of Akt in IGF-1 signaling [63]. In 2012 and 2014, we and other group further showed that EGF-mediated Akt activation also requires K63-linked ubiquitination of Akt, which is induced by Skp2 or TRAF4 [64, 65]. Experiments using either xenograft or genetic mouse models illustrated that deficiency in Skp2, TRAF6 or TRAF4 results in suppression of cancer development in vivo, pointing to the critical role of distinct E3 ubiquitin ligases in Akt-mediated biological functions and cancer progression. Notably, ablation of Skp2 mitigates the elevation of Akt phosphorylation and activation in tumors tissues driven by overexpressed Her2/Neu oncoprotein [64]. Altogether, these studies suggest that various extracellular signals utilize distinct E3 ubiquitin ligases to orchestrate Akt signaling activation. Future studies are needed to define the mechanism by which distinct E3 ubiquitin ligases are selectively utilized for Akt activation by diverse extracellular signals. Since Akt signaling pathways are also activated by other growth factors and cytokines, such as FGF, PDGF and insulin, it will be interesting to determine whether K63-linked ubiquitination of Akt is a general mechanism for Akt signaling activation beyond IGF-1, EGF and IL-1. If it is the case, what E3 ubiquitin ligases are also engaged in Akt activation other than Nedd4, Skp2, TRAF4 and TRAF6? Even under stimulation by the same ligand, Akt activity can be modulated by more than one E3 ligase. What is the crosstalk between these E3 ligases in Akt activation? It would be interesting to know how they work together: redundantly, complementarily or distinctly?

The ubiquitination can be reversed by deubiquitinating enzymes (DUBs). Recently, two independent studies reported that the tumor suppressor CYLD can function as a DUB for Akt. CYLD can terminate Akt activity by removing Akt K63-linked ubiquitin chains induced by different inducers, including growth factors and lung injury stimuli [66, 67]. Thus, CYLD serves as a negative regulator for Akt-mediated tumorigenesis and lung fibrosis. These reports suggest that the K63-linked ubiquitination is a universal mechanism for Akt regulation at least beyond growth factor signaling. These findings underscore the crucial role of cycles of ubiquitination and deubiquitination in Akt plasma membrane localization and activation. Several questions remain to be answered. For examples, what mechanism causes CYLD to switch off Akt from the ubiquitinated (active) to deubiquitinated form (inactive)? Is CYLD the sole actor or are there more DUBs responsible for Akt deubiquitination and regulation?

Besides ubiquitination, multiple frequent posttranslational modifications occur at lysine residues, such as SUMOylation and acetylation. Li and colleagues systematically screened 34 lysine residues on Akt to determine which sites are engaged in Akt activation by analyzing phosphorylation of Akt and its downstream target, GSK3β. This study supports the previous finding that the K14 residue, modified by K63-linked ubiquitination, is essential for Akt phosphorylation and activation [68]. Consistent with previous observations, mutation of K179, the ATP-binding site, impairs the activation of GSK3β without impact on the serine/threonine phosphorylation of Akt [68]. Other than residues K14 and K179, replacement of lysine with arginine at K168, 183, or K276 reduces the phosphorylation of GSK3β by Akt but has negligible impact on the phosphorylation state of Akt. Among these sites, K276 was identified as a major receptor site for SUMOylation, which is provoked by the SUMO E3 ligase PIAS1 and reversed by the SUMO-specific protease SENP1 [68]. Another biochemical analysis revealed that K301, like K276, is also a prevalent SUMO attachment site on Akt. Moreover, double mutation on K276 and K301 residues of Akt was found to attenuate Akt-regulated cellular processes independently of Akt phosphorylation [69]. Even though SUMOylation of Akt is critical for Akt activation, the mechanism accounting for its activation remains to be determined. It’s worth mentioning that SUMOylation can occur on all three Akt isoforms while K63-linked ubiquitination can be detected in Akt1 and Akt2 but not Akt3. These observations imply that K63-linked ubiquitination and SUMOylation may activate Akt through different mechanisms—at least in Akt3.

Acetylation is another lysine modification that can negatively regulate Akt activity [70]. It has been demonstrated that the level of Akt acetylation induced by IGF-1 or insulin inversely correlates with Akt phosphorylation and signaling [70]. p300 and p300/CBP-associated factor (PCAF) are the aceyltransferases that induce Akt acetylation and inactivation, whereas sirtuin 1 (SIRT1) deacetylates Akt to promote Akt binding to PIP_3_ and consequent Akt activation [70]. Consistent with these observations, SIRT1 deficiency compromises Akt participation in cell survival, proliferation and tumorigenesis. Mass spectrometry analysis revealed that Akt is acetylated at K14 and K20 within its PH domain. While the Akt acetylation-defective mutant, K20R, exhibits increased PIP_3_ binding and Akt membrane localization, the K20Q mutant that mimics constitutive Akt acetylation increases Akt activity, recapitulating the effect of SIRT1 deficiency. However, the other acetylation defective Akt mutant (K14R) does not enhance Akt phosphorylation as expected and activation due to the fact that it also affects K63-linked Akt ubiquitination. Thus, it is likely that the loss of K63-linked ubiquitination offsets enhanced Akt membrane translocation and activation by the loss of Akt acetylation. While several lysine modifications on Akt have been uncovered, more studies will be required to decipher the potential crosstalk among diverse lysine modifications on Akt.

### Glycosylation of Akt

O-GlcNAcylation is a reversible process, which modifies serine/threonine residues of proteins with a specific glycan: β-N-acetylglucosamine. Some studies have demonstrated that elevated protein O-GlcNAcylation by overexpression of O-GlcNAc transferase or inhibitors of O-GlcNAcases results in decreased Thr 308 phosphorylation of Akt and thus insulin resistance [71, 72]. However, whether Akt can be the target for O-GlcNAcylation was not characterized in these studies. Subsequent study from Soesanto and colleagues reports that Akt itself can undergo O-GlcNAcylation [73]. Geng’s and Gong’s groups later demonstrated that O-GlcNAcylation of Akt inhibits its Thr 308 phosphorylation in various cell types [74, 75]. Geng’s group further mapped the O-GlcNAcylation sites of Akt and illustrated that O-GlcNAcylation occurs at Thr305 and Thr312. Intriguingly, O-GlcNAcylation of Akt attenuates Akt phosphorylation at Thr308 and Akt-mediated biological functions by blocking the binding of Akt to PDK1. Although O-GlcNAcylation of Akt on Ser126 and Ser129 was also reported, the progress toward understanding the functional significance of O-GlcNAcylation on these two sites was hindered by the fact that Ser126 and Ser129 are also Akt phosphorylation sites. Experiments comparing the effects of O-GlcNAcylation versus phosphorylation of Akt on Ser126 and Ser129 are necessary to determine the predominant and functional relevance of these modifications on these sites. Furthermore, the interplay between of O-GlcNAcylation and phosphorylation on Ser126 and Ser129 in Akt regulation remains to be elucidated.

### Akt signaling in human cancer

Although mutations in Akt are rarely found, Akt signaling is one of the most frequently hyperactivated pathways in many human cancers. Several mechanisms accounting for this aberrant activation have been proposed. These include mutation or amplification of PIK3C gene that encodes PI3K, loss of PTEN function and activation of RTKs. Thus, development of inhibitors targeting PI3K-Akt pathway has received enormous attention in the past decade [76, 77]. The antitumor activities of specific PI3K or dual PI3K/mTOR inhibitors have been reported in several preclinical models [78–81]. In theory, the dual PI3K/mTOR inhibitors should lead to the maximal inhibition of PI3K-Akt pathway as they can simultaneously target PI3K, mTORC1 and mTORC2. The potential therapeutic benefits in patients and adverse effects that may come with these inhibitors are currently being evaluated in a number of clinical trials (the progress of the specific PI3K or dual PI3K/mTOR inhibitors in clinical trials is comprehensively reviewed by C. Porta) [82]. Among these, NVP-BEZ235, an ATP-competitive dual inhibitor for PI3K and mTOR, is currently in phase I/II clinical trials for advanced solid tumors. Although the clinical benefits of NVP-BEZ235 are being assessed, multiple resistance mechanisms are already known in prostate, breast and renal cell carcinomas (RCC) cells, among others. Inhibition of the PI3K/Akt pathway by NVP-BEZ235 activates androgen receptor signaling by relieving feedback inhibition of Her2 kinases [83, 84], thus maintaining tumor cell growth in PTEN-deficient murine prostate cancer models. Similarly, use of NVP-BEZ235 in breast and RCC cells induces feedback regulations to sustain cell survival via ERK- and the FoxO/mTORC2-dependent pathways, respectively [85, 86]. These findings highlight that combination therapies of NVP-BEZ235 with inhibitors that target the respective resistance pathways prevalent in each cellular context should be considered in order to maximize the therapeutic efficacy in the clinic setting. Further studies are needed to unravel comprehensive resistance mechanisms that cancer cells may evolve to survive in the presence of NVP-BEZ235 or other PI3K inhibitors. This information will provide rationale for combination therapy in cancer patients.

Membrane translocation is known to be prerequisite for Akt phosphorylation and signal activation. Perifosine is a PH domain inhibitor that targets Akt activity via perturbing membrane translocation of Akt and has been proven as the most clinically advanced Akt inhibitor [87]. However, perifosine along with other pH domain, lipid-based Akt inhibitors such as PX-316 [88], inhibit not only Akt but also other pH domain containing proteins. Hence, specificity and the potential for severe side effects, like hemolysis, are common issues needed to be resolved for the class of PH domain Akt inhibitors. A growing body of evidence has shown that cycles of K63-linked ubiquitination of Akt provide dynamic and precise control of Akt activity by regulating its membrane localization [62–67]. The discoveries that cancer-associated Akt E17K mutant exhibits higher basal level of K63-linked ubiquitination than wild-type Akt provide an underlying mechanism through which the Akt E17K mutant acquires increased activity and oncogenic potentials in cancer [47, 62, 63]. This observation highlights the physiological relevance of K63-linked ubiquitination in Akt regulation and further suggests that E3 ligases responsible for growth factor induced K63-linked ubiquitination of Akt can serve as a new class of therapeutic targets in cancer treatment [89]. Indeed, deficiency of Akt E3 ligases including Skp2, TRAF6 and TRAF4 suppresses tumor growth in vivo. In agreement with these genetic findings, the specific Skp2 inhibitor against its E3 ligase activity, compound 25, downregulates Akt phosphorylation and represses tumor growth in pre-existing cancer xenografts [90, 91]. These studies open an avenue to treat human cancers by targeting E3 ligases for K63-linked Akt ubiquitination.

### Conclusion and perspectives

**Figure 1:**
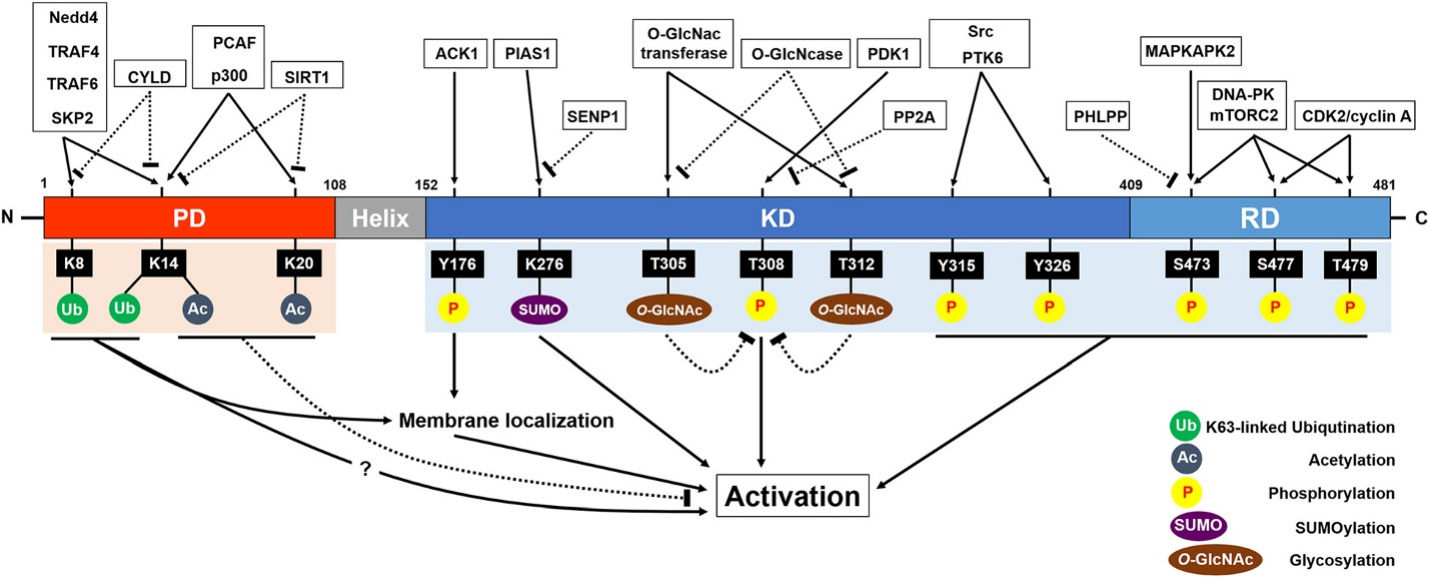
**Schematic representation of post-translational modifications of Akt.** Domain structure and post-translational modification sites of Akt are shown together with their regulating proteins. PH (Pleckstrin homology domain), KD (Kinase domain), and RD (Regulatory domain). Numbers indicate amino acid position.

Research on Akt regulation by serine/threonine phosphorylation has taken center stage for years, and Akt and its upstream regulators, like PI3K, have been targeted for cancer therapies. The discovery of other posttranslational modifications such as ubiquitination, SUMOylation, acetylation and glycosylation adds further complexity to the diverse regulatory networks controlling Akt signaling activation (Figure 1). Even though aberrant deregulation of PI3K and PTEN activity is a prevalent cause for Akt hyperactivation in human cancers, mechanisms other than PI3K/PTEN deregulation have emerged [47–49]. Recent advances reveal that K63-linked ubiquitination, SUMOyation and perhaps tyrosine phosphorylation can contribute to Akt signaling activation in a PI3K-independent fashion, indicating that these modifications may be alternative mechanisms accounting for aberrant Akt hyperactivation in human cancers. Thus, the identification of proteins responsible for these modifications will provide important insights for cancer treatment.

Accumulating evidence illustrates that different Akt isoforms are responsible for their diverse biological functions [92–94]. In particular, Akt1 and Akt2 seem to have opposing roles in cancer metastasis despite the similarity of their activation mode by PI3K stimulation [95–98]. This may explains the poor efficacy of PI3K inhibitors in advanced human cancers. It also suggests that deeper understanding of the roles of PTMs on the activation of different Akt isoforms over the course of cancer progression and metastasis could be beneficial for therapeutic outcomes. Limited evidence has been presented that K63-linked ubiquitination and SUMOylation can efficiently modify both Akt1 and Akt2 [62, 64, 69], suggesting that neither modification is the major contributor for the diverse functions of Akt1 and Akt2 in metastasis. Further effort is required to define what PTMs may be associated with the activation of distinct Akt isoforms. This knowledge will offer an opportunity to develop specific inhibitors against each Akt isoform.

Despite the enormous progress made for the role of PTMs in Akt regulation, several outstanding questions remain to be addressed. While both K63-linked ubiquitination and SUMOylation have been shown to drive Akt localization to either membrane and/or nucleus, the underlying mechanism dictating Akt trafficking remains unknown. Do K63-linked ubiquitination and SUMOylation employ the same regulatory machinery for Akt trafficking even though modifications occur at different sites? What determines the biological consequences when distinct modifications occur on the same residues of Akt? For instance, both K63-linked ubiquitination and acetylation can occur at K14 upon IGF1 stimulation. How do they work differently to coordinately regulate Akt activation in response to IGF-1? Another example is how phosphorylation and O-GlcNAcylation of Akt can occur on the same sites of Akt (Ser126 and Ser129). Are these two modifications mutually exclusive or interdependent? Comprehensive understanding of physiologically importance of crosstalk between PTMs in Akt regulation may provide better strategies for the fight against cancer.
